# P-749. Prognostic Determinants in Patients with Necrotizing Fasciitis in a Referral Hospital in Nicaragua

**DOI:** 10.1093/ofid/ofae631.945

**Published:** 2025-01-29

**Authors:** Francisco Hernández-Hernández, Guillermo D Porras-Cortés

**Affiliations:** Hospital Dr. Fernando Vélez Paiz, Altagracia, Rivas, Nicaragua; Hospital Dr. Fernando Vélez Paiz, Altagracia, Rivas, Nicaragua

## Abstract

**Background:**

Necrotizing fasciitis is a serious condition in which timely diagnosis and therapeutic approaches, both medical and surgical, play an essential role in prognosis. In Nicaragua, the prognostic determinants for this clinical condition in different hospital institutions have not been formally evaluated. The aim of this study was to establish factors that could be determinant for the prognosis of patients with necrotizing fasciitis in a national referral hospital.

**Table 1.**
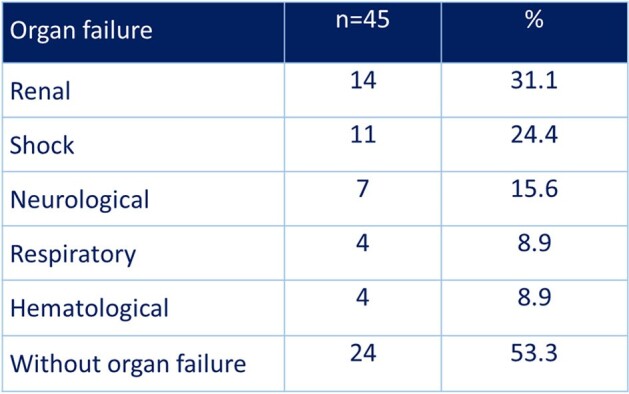
Organ Failure at the Admission in Patients with Necrotizing Fasciitis

**Methods:**

This is a retrospective, cross-sectional, analytical study. It was conducted with patients over 15 years of age with a diagnosis of necrotizing fasciitis hospitalized between January 201 and December 2023 in Dr. Fernando Vélez Paiz Hospital in Managua, Nicaragua. Clinical factors such as comorbidities or organ failure on admission, as well as the LRINEC score, and the times of administration of the first dose of antibiotic and surgical intervention were analyzed and correlated with the clinical outcome of the patients.

**Table 2.**
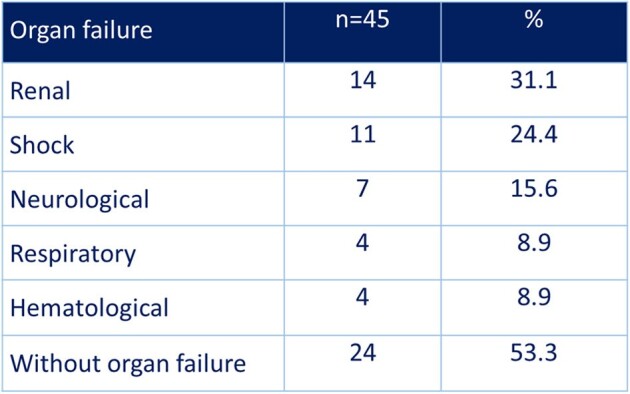
Potentials Prognosis Determinants in Necrotizing Fasciitis that were Evaluated

**Results:**

A total of 45 patients were included in the study, 60% of whom were women. The mean age was 56.0 ± 14.6 years-old. 86.7% of patients had at least one comorbidity. At the time of diagnosis, 31.1% of patients had renal insufficiency, 24.4% had shock, and 15.6% had altered state of consciousness (Table 1). 25 patients died for a mortality rate of 44.4%. The mean time from suspected diagnosis and administration of the first dose of antibiotics was 2.5 ± 1.4 hours (Table 2). Only 20% of patients received the first dose of the antibiotic at the first hour. 17.8% of patients underwent surgery within 6 hours of diagnosis. There was no association between prognosis and the time of initiation of antibiotic therapy and the time of initiation of surgical treatment after diagnosis of necrotizing fasciitis, probably due to a problem of sample size. 60% of the deceased had LRINEC ≥ 8 points, while only 28% in the group of survivors, OR(95%CI): 3.85 (1.10-13.46) (Table 3).

**Table 3.**
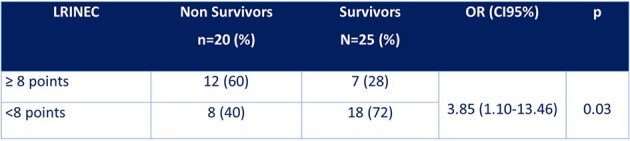
LRINEC and Mortality in Patients with Necrotizing Fasciitis

**Conclusion:**

Patients with necrotizing fasciitis mostly have comorbidity, mainly diabetes, and almost a quarter of them have shock and a third have renal failure. A LRINEC score greater than or equal to 8 points significantly increases the risk of death in this type of patient.

**Disclosures:**

**All Authors**: No reported disclosures

